# Effects of Anterior Cruciate Ligament Reconstruction on Gluteal Muscle Volume

**DOI:** 10.51894/001c.143936

**Published:** 2025-09-30

**Authors:** Claire Bischel

**Affiliations:** 1 College of Osteopathic Medicine Michigan State University

## 23

### INTRODUCTION

Anterior cruciate ligament (ACL) injuries lead to quadriceps atrophy and altered biomechanics despite ACL reconstruction surgery (ACLR). Previous research has confirmed that decreased proximal muscle performance can lead to altered biomechanics, thereby putting the knee at risk for ACL injury or reinjury. However, less is known about the role of proximal hip muscles post-ACLR.

### HYPOTHESIS

The purpose of this study was to determine whether the proximal gluteal muscles in individuals post-ACLR demonstrate muscle atrophy. We hypothesized that at both 6- and 12-months post-ACLR, significant atrophy will be observed.

### METHODS

11 participants (n=9 females) with unilateral ACLR participated in this IRB-approved study. All subjects underwent axial magnetic resonance imaging (MRI) scans (iliac crest to ankle joint) at 6 months and 12 months post-ACLR. In order to calculate muscle volume, cross-sectional area of the gluteus maximus (GMAX), medius (GMED) and minimus (GMIN) were identified and traced on each axial MRI slice using Horos imaging software (horosproject.org). Muscle volume was calculated as the cross-sectional area of each slice will be multiplied by the thickness of the image (e.g., 10 mm) and summed. Comparisons between the ACLR vs. uninvolved limb for each muscle volume at each time interval post-ACLR were assessed using dependent t-tests. All statistical analyses were conducted using SPSS with significance set at p≤0.05. Trends were considered when p-values between 0.05 and 0.10.

### RESULTS

At both the 6- and 12-month timepoints, there were no significant differences in any of the gluteal muscles (GMAX, GMED, GMIN) between the involved and uninvolved limbs (all p>0.05). However, a trend (p=0.07) was observed at 6 months, with GMAX being larger on the involved limb (951.7 ± 162.3 cm3) compared to the uninvolved limb (926.4 ± 168.7 cm3).

### CONCLUSION

Given that the increased volume of GMAX on the involved limb was only observed at the 6-month time point, reliance on GMAX may be a compensatory mechanism used to offload the quadriceps that is restricted to the early phases of recovery. Future research investigating the biomechanical response to ACLR and its effects on gluteal musculature could help optimize recovery and improve long-term outcomes.

